# *S*-Fluorenylmethyl protection of the cysteine side chain upon *N*^α^-Fmoc deprotection

**DOI:** 10.3762/bjoc.8.242

**Published:** 2012-12-10

**Authors:** Johannes W Wehner, Thisbe K Lindhorst

**Affiliations:** 1 Otto Diels Institute of Organic Chemistry, Christiana Albertina University of Kiel, Otto-Hahn-Platz 3/4, 24098 Kiel, Germany

**Keywords:** Fmoc protecting group, glycoamino acids, *N*-Fmoc→*S*-Fm transprotection, protecting groups

## Abstract

Deprotection of an *N*^α^-Fmoc-protected glycocysteine derivative **7** with an excess of morpholine unexpectedly led to the fluorenylmethyl-protected thioether **8** in high yield. The suggested mechanism for this reaction comprises the addition of the cysteine thiolate on the exocyclic double bond of dibenzofulvene, which is formed during Fmoc deprotection. The influence of base concentration on this transprotection reaction was studied.

## Introduction

In the course of our work on the synthesis of glycoamino acids, we have recently used L-cysteine as a scaffold for the synthesis of various glycoclusters [1–3]. This is an attractive concept, because it can be combined with solid-phase peptide synthesis (SPPS) [4–5], as well as with native chemical ligation (NCL) utilizing an *S*→*N* acyl shift [3,6–10]. In addition, glycocysteine derivatives can be easily converted into the corresponding dimers by oxidiation of the cysteine moiety into the respective cystine form.

Indeed, preparation of glycoamino acid derivatives such as **3-dimer**, an oxidized cysteine, or cystine derivative, is facile and can be realized via different synthetic routes. However, as we employed the fluorenylmethoxycarbonyl (Fmoc) protecting group for the synthesis of **3-dimer**, we observed an unexpected but high-yielding *S*-fluorenylmethyl (Fm) protection of the cysteine side chain during *N*-Fmoc deprotection. Hence, this side reaction was further investigated under different reaction conditions and the results of this study are described in this account together with a survey of the synthetic approaches to obtain **3** and **3-dimer**.

## Results and Discussion

**Scheme 1:**
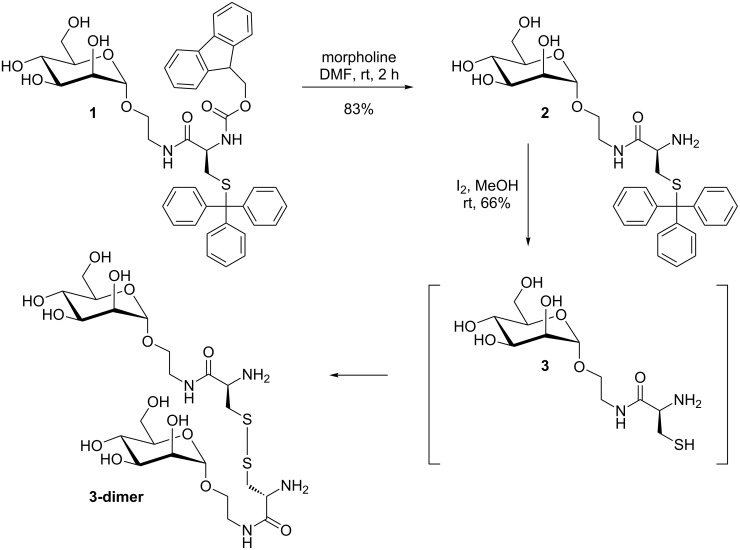
Synthesis of glycoamino acid derivative **3** and its dimer, from the known mannopyranoside **1**.

Synthesis of **3-dimer** was started from the known *N*,*S*-protected glycoamino acid derivative **1** [2], which can be obtained by peptide coupling of 2-aminoethyl α-D-mannopyranoside [11] and the corresponding protected cysteine derivative, Fmoc-Cys(Trt)-OH (Scheme 1). Fmoc-deprotection by using morpholine as the base gave the free amine **2**, and then removal of the *S*-trityl protecting group under oxidative conditions (iodine in methanol) led to the cystine derivative **3-dimer** after in situ oxidation of the intermediate free thiol in good yield.

**Scheme 2:**
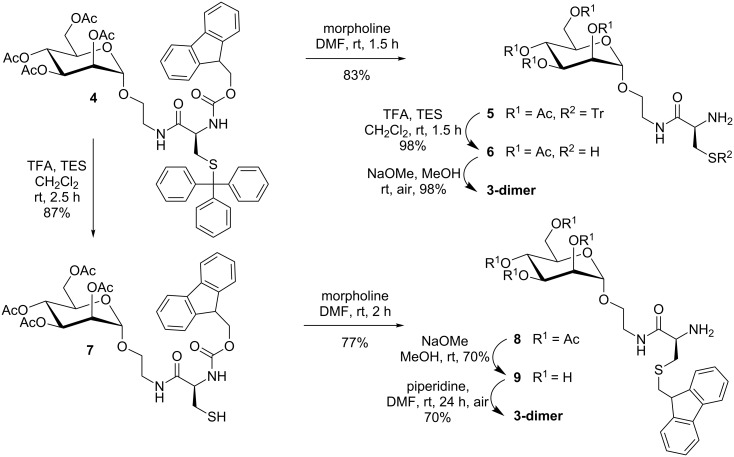
To obtain the glycocystine derivative **3-dimer** from the protected cysteine mannopyranoside precursor **4**, the sequence of deprotection steps matters. When the thiol function was deprotected first to give **7**, then *N*-Fmoc deprotection resulted in *N*-Fmoc→*S*-Fm transprotection. On the other hand, Fmoc deprotection was no problem starting from **4** with the thiol function protected.

As *O*-acetylated building blocks are often advantageous over the OH-free analogues for SPPS [2–4], our next step was to apply the synthesis outlined in Scheme 1 to the *O*-acetylated glycoamino acid derivative **4** (Scheme 2). The *O*-acetylated mannopyranside **4** can be prepared by Staudinger ligation of *O*-acetyl-protected 2-azidoethyl α-D-mannopyranoside [11] and the cysteine derivative Fmoc-Cys(Trt)-OH as described earlier [1]. Then, a sequence of Fmoc-deprotection, leading to **5** and acidic removal of the trityl group by using TFA and triethylsilane (TES) as cation scavenger [12] yields the *O*-acetylated glycoamino acid derivative **6** together with its respective disulfide (not shown in Scheme 2), and de-*O*-acetylation under Zemplén conditions [13] furnishes the unprotected compound **3-dimer** after oxidation in air, as reported previously [3]. However, when the trityl group in **4** was removed first, thiol **7** was obtained as expected, but the following Fmoc deprotection under standard conditions [14], employing 6 equiv of morpholine in DMF, unexpectedly led to the *S*-fluorenylmethyl (Fm)-protected glycoamino acid **8** in 77% yield (Scheme 2). The anticipated glycoamino acid derivative **6** was not obtained. De-*O*-acetylation of **8** gave mannopyranoside **9** with maintained fluorenylmethyl protection at the sulfur atom.

**Figure 1:**
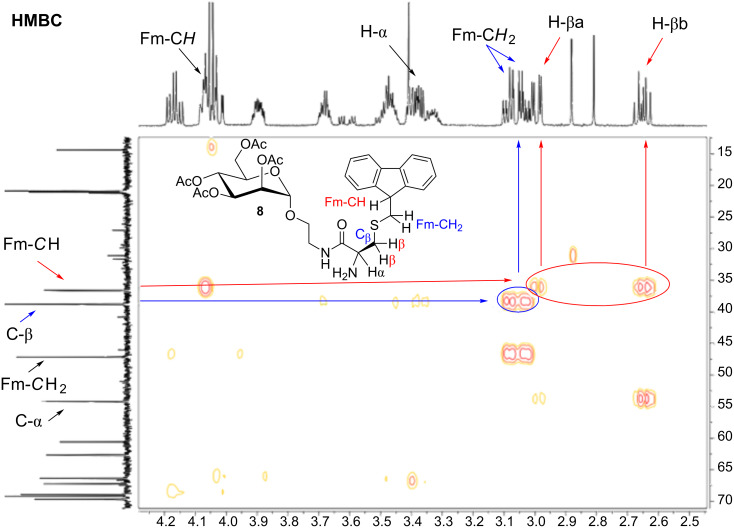
In the ^1^H/^13^C HMBC NMR spectrum of the *S*-Fm-protected glycoamino acid derivative **8**, protecting-group rearrangement is confirmed by the cross peaks between C-β and the Fm-C*H_2_* protons (in blue) and between Fm-*C*H and the H-βa,β protons (in red).

The structure of the *S*-Fm-protected glycoamino acid derivative **8** could be unequivocally confirmed by NMR analysis and MALDI–TOF mass spectrometry. The HMBC NMR spectrum of **8** (Figure 1) clearly indicates that the formerly unprotected thiol group of the cysteine side chain of **7** became protected by a fluorenylmethyl (Fm) moiety. This is indicated by the respective cross peaks between C-β and the Fm-C*H_2_* protons on one hand and between Fm-*C*H and the H-βa and H-βb protons on the other hand.

**Scheme 3:**
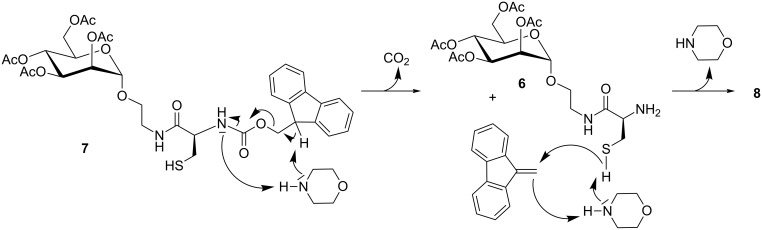
Proposed mechanism for the formation of *S*-Fm-protected **8** from *N*-Fmoc-protected **7** according to Rich and co-workers [16].

*N*-Fmoc→*S*-Fm transprotection was reported earlier by Katritzky et al. for cysteine peptides [15]. In this case, DBU was employed as the base and the reaction was conducted in dry THF at 0 °C for 15 min to give the rearrangement products in 69–87% yield. The mechanistic rational proposed by the Katrizky group is based on a report by Rich et al., where the influence of the employed base on *N*-Fmoc→*S*-Fm transprotection was studied [16]. According to the proposal provided by Rich et al., formation of the cysteine derivative **7** can be explained by an elimination reaction of the E1cb type, in which *N*^α^ is deprotected with the release of carbon dioxide and formation of dibenzofulvene (Scheme 3). Then, presumably, the cysteine thiol is deprotonated by morpholine. Since the nucleophilicity of the so formed thiolate is higher than that of morpholine or the generated primary *N*^α^ amino group, the in situ generated fulvene is quenched by the cysteine thiolate, leading to *S*-Fm-protected **8**.

Next, we have investigated different reaction conditions for the conversions of **7** and **9** with piperidine, which is the common base used in SPPS. Deprotection of the *S*-Fm-protected glycoamino acid derivative **9** was possible by using 50% piperidine in DMF at room temperature to yield the fully deprotected disulfide **3-dimer** in 70% yield (Table 1). Separately, **7** was treated with different concentrations of piperidine. When 0.1 equiv of piperidine in DMF was employed, only about 20% of the fully deprotected mannopyranoside **6** was obtained, whereas over 40% of the *N*→*S*-transprotected product **8** was formed. Concurrently, the doubly *N*,*S*-protected derivative **10** was identified as an additional product (Table 1). When 1 equiv of piperidine was employed, the overall yield of **8** and **6** was increased, while no doubly *N*,*S*-protected compound **10** was detected. However, transprotection occurred with 66%. Further increase of the piperidine concentration to 6 equiv led to 47% transprotection, whereas when the same amount of morpholine was used at 100 mM concentration, 62% transprotection occurred. Standard SPPS Fmoc deprotection conditions with 20% piperidine in DMF, however, led to complete deprotection of **7** and a high combined yield of the desired glycoamino acid derivative **6** and its respective cystine dimer.

**Table 1:** Product analysis of deprotection reactions under different basic reaction conditions.^a^

Starting material/ used base^b^	Products		

**9**/50% piperidine^c^	70% **3-dimer**		

	**8**	**6** and its disulfide	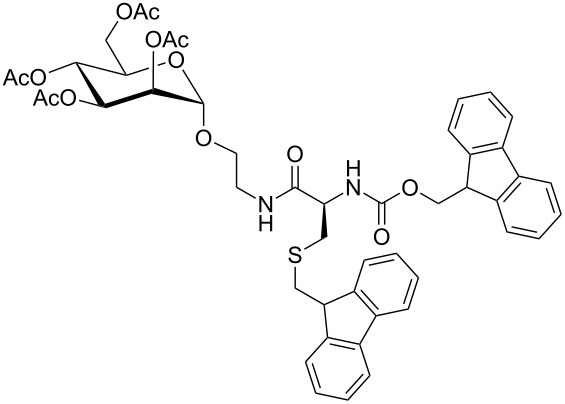 **10**

**7**/0.1 equiv piperidine	43%	22%	22%^d^
**7**/1 equiv piperidine^a^	66%	32%	–
**7**/6 equiv piperidine	47%	46%	–
**7**/6 equiv morpholine^e^	62%	35%	–
**7**/20% piperidine	–	93%	–

^a^Only products obtained with yields >5% were isolated and analyzed; ^b^reaction conditions: **7** (50 mg in dry DMF) was reacted at a concentration of 100 mM for 2 h at rt under a nitrogen atmosphere; ^c^this reaction was carried out at a concentration of 57.5 mM; ^d^confirmed by MALDI–TOF mass spectrometry; ^e^6 equiv morpholine at a concentration of 173 mM led to 77% **8** (see above).

These experiments show that the amount of base is crucial to avoid *N*-Fmoc→*S*-Fm transprotection during *N*-Fmoc removal. The problem of transprotection can be solved by choosing the appropriate base at appropriate concentrations.

## Conclusion

Glycocysteine/cystine derivatives are versatile building blocks for the synthesis of a wide variety of glycoconjugates, which are thus accessible by, among other methods, NCL or SPPS. In addition, mannosidic glycoamino acids are of interest as ligands for mannose-specific lectins in solution [17] as well as for the fabrication of glycoarrays on solid support [10,18–19]. Moreover, we are interested to advance glycoamino acid deriatives such as **3** into ligands that can be “switched” between two states by oxidation/reduction. A key step, when glycoamino acids are further conjugated, is Fmoc protection and deprotection. Here, we have shown an *N*-Fmoc→*S*-Fm transprotection reaction, which can occur upon *N*-Fmoc deprotection, and how it can be controlled under different basic reaction conditions. Though this type of transprotection reaction has been described before, here the first example with glycoamino acid derivatives is reported. The results of our study can be utilized wherever glycocysteine derivatives are employed in the synthesis of glycoclusters or glycopeptides, for example, in the context of an orthogonal protection/deprotection approach.

## Experimental

### General methods

Commercially available starting materials and reagents were used without further purification unless otherwise noted. Glycoamino acid derivatives **1** [1], **2** [2], **3** [3], **4** [2], **5** [2] and **6** [3] were prepared according to the literature. Anhydrous DMF was purchased; other solvents were dried for reactions or distilled for chromatography. Air- or moisture-sensitive reactions were carried out under an atmosphere of nitrogen. Thin-layer chromatography was performed on silica-gel plates (GF 254, Merck). Detection was effected by UV irradiation and subsequent charring with 10% sulfuric acid in EtOH followed by heat treatment. Flash chromatography was performed on silica gel 60 (230–400 mesh, particle size 0.040–0.063 mm, Merck). Preparative MPLC was performed on a Büchi apparatus by using a LiChroprep RP-18 column (40–60 µm, Merck) for reversed-phase silica-gel chromatography. ^1^H and ^13^C spectra were recorded on Bruker DRX-500 and AV-600 spectrometers at 300 K. 2D NMR experiments (^1^H/^1^H COSY, ^1^H/^13^C HSQC and ^1^H/^13^C HMBC) were performed for full assignment of the spectra. Chemical shifts are relative to internal TMS (^1^H: δ 0.00 ppm) or were calibrated relative to solvent peaks of CHCl_3_ (^13^C: δ 77.0 ppm), MeOH (^1^H: δ 3.31 ppm; ^13^C: δ 49.0 ppm), or H_2_O (δ 4.65 ppm). For peak assignment, atoms were numbered according to conventions for carbohydrate and amino-acid nomenclature. The abbreviation “Fmoc” refers to fluorenylmethoxycarbonyl and “Fm” to fluorenylmethyl. ESI mass spectra were recorded on a Mariner ESI-TOF 5280 (Applied Biosystems) instrument and MALDI-MS measurements on a MALDI-TOF-MS-Biflex III (Bruker) instrument by using 2,5-dihydroxybenzoic acid (DHB) or α-cyano-4-hydroxycinnamic acid (CCA) as matrix. IR-spectroscopic measurements were recorded on a FTIR spectrometer Paragon 1000 (Perkin-Elmer) by using a Golden-Gate diamond-ATR unit with a sapphire stamp. Optical rotations were measured on a Perkin-Elmer 241 polarimeter (Na-D-line: 589 nm, length of cell 1 dm).

#### L-Cystine-bis[2-(α-D-mannopyranosyloxy)ethyl]amide (**3-dimer**)

The *S*-Fm-protected glycoamino acid derivative **9** (290 mg, 575 μmol) was dissolved in dry DMF (5 mL), and then piperidine (5 mL) was added, and the reaction mixture was stirred for 24 h at ambient temperature under a nitrogen atmosphere. Then the reaction mixture was concentrated in vacuo, and the crude product was purified by two subsequent RP-MPLC purification steps: (i) RP-18, A = water, B = methanol, B: 10% → 50%, 60 min; (ii) RP-18, A = water, B = ethanol, B: 95% → 20%, 60 min). After lyophilization, the title compound **3** (130 mg, 200 μmol, 70% based on monomeric starting material) was obtained. *R*_f_ 0.74 (RP-18, MeOH); [α]_D_^23^ +15 (*c* 0.6, MeOH); IR (ATR) 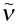 3270, 2919, 1648, 1546, 1409, 1200, 1130, 1050, 963, 609, 458 cm^−1^; ^1^H NMR (600 MHz, D_2_O) δ 4.91 (d, ^3^*J*_1,2_ = 1.8 Hz, 2H, 2 H-1), 3.99 (dd, ^3^*J*_1,2_ = 1.8 Hz, ^3^*J*_2,3_ = 3.4 Hz, 2H, 2 H-2), 3.92 (dd, ^3^*J*_5,6_ = 4.3 Hz, ^2^*J*_6,6’_ = 12.0 Hz, 2H, 2 H-6), 3.90–3.88 (m, 2H, 2 H-α), 3.87–3.83 (m, 4H, 2 H-3, 2 man-OC*H*H), 3.78 (dd, ^3^*J*_5,6_ = 5.9 Hz, ^2^*J*_6,6’_ = 12.0 Hz, 2H, 2 H-6’), 3.69–3.65 (m, 6H, 2 H-4, 2 H-5, 2 man-OC*H*H), 3.58 (m_c_, 2H, 2 man-OCH_2_C*H*H), 3.45 (ddd ~ tdd, ^3^*J* = 4.0 Hz, ^3^*J* = 6.7 Hz, ^2^*J* = 14.6 Hz, 2H, 2 man-OCH_2_CH*H*), 3.15–3.06 (m, 4H, 2 H-β, 2 H-β’) ppm; ^13^C NMR (150 MHz, D_2_O) δ 175.5 (2 *C*(O)C-α), 101.5 (2 C-1), 74.6 (2 C-5), 72.3 (2 C-3), 71.8 (2 C-2), 68.5 (2 C-4), 67.5 (2 man-O *C* H_2_CH_2_), 62.7 (2 C-6), 55.2 (2 *C*-α), 42.7 (2 *C*-β), 40.9 (2 man-OCH_2_* C* H_2_) ppm; HRMS–ESI (*m*/*z*): calcd for C_22_H_42_N_4_NaO_14_S_2_, 673.2031; found, 673.2183.

#### Procedure for the preparation of **3-dimer** by iodine oxidation of **2** (Scheme 1)

Manoside **2** (500 mg, 880 µmol) was dissolved in methanol (2 mL), and then iodine (334 mg, 2.62 mmol) dissolved in methanol (9 mL) was added dropwise. After being stirred for 45 min at ambient temperature, the reaction was quenched with saturated sodium thiosulfate solution (3 mL), and solvents were removed under reduced pressure. The crude product was subjected to size-exclusion chromatography (LH-20, methanol) yielding **3-dimer** (377 mg, 580 µmol, 66%) as a colorless lyophylisate. Analytical data are according to the literature [3].

#### *N*-(Fluoren-9-ylmethoxycarbonyl)-L-cysteine-[2-(2,3,4,6-tetra-*O*-acetyl-α-D-mannopyranosyloxy)ethyl]amide (**7**)

The *S*-protected glycoamino acid derivative **4** (1.35 g, 1.41 mmol) was dissolved in dry dichloromethane (8 mL), and then triethylsilane (1.26 mL, 7.91 mmol) and TFA (600 μL, 7.79 mmol) were added, and the reaction mixture was stirred for 2 h at ambient temperature under a nitrogen atmosphere. Then another portion of TFA (600 μL, 7.79 mmol) was added and stirring was continued for 30 min until no further conversion of **1** was observed by TLC (toluene/ethyl acetate, 1:1). The reaction mixture was concentrated under reduced pressure and repeatedly codistilled with toluene (3 × 20 mL). The crude product was purified by flash column chromatography (toluene/ethyl acetate, 2:1) yielding **7** (884 mg, 1.23 mmol, 87%) as a colorless foam. *R*_f_ 0.06 (toluene/ethyl acetate, 2:1); [α]_D_^23^ +25 (*c* 0.7, CHCl_3_); IR (ATR) 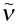 3311, 2932, 1741, 1661, 1519, 1449, 1367, 1215, 1135, 1079, 1042, 977, 910, 728, 644, 598, 463 cm^−1^; ^1^H NMR (600 MHz, CDCl_3_, TMS) δ 7.70 (d, ^3^*J* = 7.6 Hz, 2H, aryl-H_Fmoc_), 7.53 (d, ^3^*J* = 7.3 Hz, 2H, aryl-H_Fmoc_), 7.33 (dd ~ t, ^3^*J* = 7.5 Hz, ^3^*J* = 7.5 Hz, 2H, aryl-H_Fmoc_), 7.29 (dd ~ t, ^3^*J* = 7.5 Hz, ^3^*J* = 7.3 Hz, 2H, aryl-H_Fmoc_), 6.62 (br s, 1H, NHα), 5.81 (d, ^3^*J* = 8.0 Hz, 1H, man-OCH_2_CH_2_-N*H*), 5.26 (dd, ^3^*J*_2,3_ = 3.6 Hz, ^3^*J*_3,4_ = 9.5 Hz, 1H, H-3), 5.21–5.15 (m, 2H, H-2, H-4), 4.77 (br s, 1H, H-1), 4.42 (m_c_, 1H, C*H*H_Fmoc_), 4.33–4.26 (m, 2H, CH*H*_Fmoc_, H-α), 4.18 (dd, ^2^*J*_6,6’_ = 12.2 Hz, ^3^*J*_5,6_ = 5.6 Hz, 1H, H-6), 4.16 (t, ^3^*J*_2,3_ = 6.9 Hz, 1H, fluorenyl-H), 4.06–4.02 (m, 1H, H-6’), 3.94 (m_c_, 1H, H-5), 3.72 (dd, ^3^*J* = 4.4 Hz, ^2^*J* = 9.0 Hz, 1H, man-OC*H*H), 3.56–3.38 (m, 3H, man-OCH*H*C*H*_2_), 3.00 (m_c_, 1H, H-β), 2.70 (m_c_, 1H, H-β’), 2.08, 2.02, 1.95, 1.91 (each s, each 3H, 4 C(O)C*H*_3_), 1.50 (br s, 1H, S-H) ppm; ^13^C NMR (150 MHz, CDCl_3_) δ 170.7 (*C*(O)CH_3_), 170.2 (*C*(O)C-α), 170.0, 169.9, 169.8 (3 *C*(O)CH_3_), 156.1 (Fmoc-O-*C*O), 143.7, 141.4, 127.8, 127.1, 125.0, 120.0 (12 C-Ar_Fmoc_), 97.7 (C-1), 69.4 (C-2), 68.9 (C-3), 68.7 (C-5), 67.2 (*C*H_2_-Fmoc), 67.0 (man-O*C*H_2_CH_2_), 66.3 (C-4), 62.6 (C-6), 56.3 (C_α_), 47.2 (CH_2_-*C*H_Fmoc_), 39.3 (man-OCH_2_*C*H_2_), 26.8 (*C*-β), 21.0, 20.8, 20.7, 20.7 (4 C(O)*C*H_3_) ppm; HRMS–ESI (*m*/*z*): calcd for C_34_H_40_N_2_NaO_13_S, 739.2143; found, 739.2033.

#### *S*-(Fluoren-9-yl)-L-cysteine-[2-(2,3,4,6-tetra-*O*-acetyl-α-D-mannopyranosyloxy)ethyl]amide (**8**)

The *N*-protected L-cysteine derivative **7** (864 mg, 1.21 mmol) was dissolved in dry DMF (7 mL), and then morpholine (640 μL, 7.30 mmol) was added, and the reaction mixture was stirred for 2 h at ambient temperature under a nitrogen atmosphere. Then the solvent was removed under reduced pressure, the residue was dissolved in methanol, and the formed precipitate was filtered off. The filtrate was concentrated under reduced pressure and the crude product was purified by gradient flash column chromatography (cyclohexane/ethyl acetate, 1:12 → ethyl acetate/methanol, 9:1 → ethyl acetate/methanol, 3:1) yielding **8** (630 mg, 936 μmol, 77%) as a colorless foam. *R*_f_ 0.45 (ethyl acetate/methanol, 9:1); [α]_D_^23^ +14 (*c* 0.7, CHCl_3_); IR (ATR) 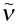 3355, 1930, 1742, 1666, 1518, 1446, 1366, 1216, 1135, 1081, 1043, 977, 911, 729, 599 cm^−1^; ^1^H NMR (600 MHz, CDCl_3_, TMS) δ 7.68 (d, ^3^*J* = 7.5 Hz, 2H, aryl-H_Fm_), 7.61 (m_c_, 2H, aryl-H_Fm_), 7.32 (dd ~ t, ^3^*J* = 7.5 Hz, 2H, aryl-H_Fm_), 7.25 (dt, ^3^*J* = 7.5 Hz, ^4^*J* = 1.0 Hz, 2H, aryl-H_Fm_), 5.27 (dd, ^3^*J*_3,4_ = 10.0 Hz, ^3^*J*_2,3_ = 3.5 Hz, 1H, H-3), 5.21–5.16 (m, 2H, H-4, H-2), 4.75 (d, ^3^*J*_1,2_ =1.2 Hz, 1H, H-1), 4.17 (dd ~ dt, ^3^*J*_5,6_ = 5.6 Hz, ^2^*J*_6,6’_ = 12.3 Hz, 1H, H-6), 4.09–4.03 (m, 2H, fluorenyl-H, H-6’), 3.90 (ddd, ^3^*J*_4,5_ = 10.1 Hz, ^3^*J*_5,6_ = 5.2 Hz, ^3^*J*_5,6’_ = 2.4 Hz, 1H, H-5), 3.68 (m_c_, 1H, man-OC*H*H), 3.52–3.44 (m, 2H, man-OCH*H*, man-OCH_2_C*H*H), 3.40–3.31 (m, 2H, H-2, man-OCH_2_CH*H*), 3.09 (dd, ^3^*J* = 6.2 Hz, ^2^*J* = 12.9 Hz, 1H, C*H*H_Fm_), 3.03 (dd, ^3^*J* = 6.4 Hz, ^2^*J* = 12.9 Hz, 1H, CH*H*_Fm_), 2.99 (dd, ^3^*J* = 3.9 Hz, ^2^*J* = 13.7 Hz, 1H, H-β), 2.65 (dd ~ dt, ^3^*J* = 8.9 Hz, ^2^*J* = 13.7 Hz, 1H, H-β’), 2.07, 2.01, 1.97, 1.92 (each s, each 3H, 4 C(O)C*H*_3_) ppm; ^13^C NMR (125 MHz, CDCl_3_) δ 173.6 (*C*(O)C-α), 170.6, 170.1, 170.0, 169.7 (4 *C*(O)CH_3_), 145.8, 141.1, 127.6, 127.0, 124.7, 119.9 (12 C-Ar_Fm_), 97.6 (C-1), 69.5 (C-2), 69.0 (C-3), 68.7 (C-5), 67.0 (man-O*C*H_2_CH_2_), 66.1 (C-4), 62.4 (C-6), 54.0 (*C*-α), 46.9 (*C*H_2_-Fm), 38.7 (*C*-β), 38.6 (man-OCH_2_*C*H_2_), 36.4 (CH_2_-*C*H_Fm_), 21.0, 20.9, 20.7, 20.7 (4 C(O)*C*H_3_) ppm; HRMS–ESI (*m*/*z*): calcd for C_33_H_41_N_2_O_11_S, 673.24; found, 673.21; MALDI–TOF–MS (CCA) *m*/*z* 673.73.

#### *S*-(Fluoren-9-yl)-L-cysteine-[2-(α-D-mannopyranosyloxy)ethyl]amide (**9**)

The *O*-acetylated glycoamino acid derivative **8** (600 mg, 892 μmol) was dissolved in dry methanol (8 mL), and then freshly prepared sodium methanolate solution (200 μL, 1 M in MeOH) was added, and the reaction mixture was stirred overnight at ambient temperature under a nitrogen atmosphere. Then acidic ion-exchange resin (Amberlite IR 120) was added, and the mixture was stirred for 5 min to allow neutralization of the solution. After filtration of the resin, the solvent was removed under reduced pressure, and the crude product was purified on silica gel (ethyl acetate/ethanol, 7:1) yielding **9** (316 mg, 626 μmol, 70%) as a colorless foam. *R*_f_ 0.24 (ethyl acetate/methanol,10:1); IR (ATR) 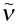 3286, 2919, 1660, 1409, 1298, 1027, 738, 501 cm^−1^; ^1^H NMR (600 MHz, CD_3_OD) δ 7.81 (d, ^3^*J* = 7.5 Hz, 2H, aryl-H_Fm_), 7.76 (dd, ^3^*J* = 4.4 Hz, ^3^*J* = 7.5 Hz, 2H, aryl-H_Fm_), 7.34 (t, ^3^*J* = 7.5 Hz, 2H, aryl-H_Fm_), 7.33 (t, ^3^*J* = 7.5 Hz, 2H, aryl-H_Fm_), 4.80 (d, ^3^*J*_1,2_ = 1.5 Hz, 1H, H-1), 4.17 (t, ^3^*J*_1(Fm),2(Fm)_ = 6.3 Hz, 1H, fluorenyl-H), 3.87 (dd, ^3^*J*_5,6_ = 2.3 Hz, ^2^*J*_6,6’_ = 11.7 Hz, 1H, H-6), 3.85 (dd, ^3^*J*_2,3_ = 3.4 Hz, ^2^*J*_1,2_ = 1.8 Hz, 1H, H-2), 3.79 (ddd, ^3^*J* = 6.4 Hz, ^3^*J* = 4.5 Hz, ^2^*J* = 10.7 Hz, 1H, man-OC*H*H), 3.75–3.70 (m, 2H, H-3, H-6’), 3.63 (dd, ^3^*J*_3,4_ = 9.2 Hz, ^3^*J*_4,5_ = 9.6 Hz, 1H, H-4), 3.60–3.54 (m, 2H, H-5, man-OCH*H*), 3.50–3.43 (m, 3H, H-α, man-OCH_2_C*H*_2_), 3.17 (ddt, ^4^*J* = 0.7 Hz, ^3^*J* = 6.3 Hz, ^2^*J* = 13.0 Hz, 2H, C*H*_2 Fm_), 2.90 (ddd, ^4^*J* = 0.7 Hz, ^3^*J* = 5.6 Hz, ^2^*J* = 13.6 Hz, 1H, H-β), 2.74 (dd, ^3^*J* = 7.2 Hz, ^2^*J* = 13.6 Hz, 1H, H-β’) ppm; ^13^C NMR (150 MHz, CD_3_OD) δ 175.9 (*C*(O)C-α), 147.4, 142.5, 128.6, 128.1, 126.0, 120.8 (12 C-Ar_Fm_), 101.7 (C-1), 74.8 (C-5), 72.6 (C-3), 72.1 (C-2), 68.7 (C-4), 67.2 (man-O*C*H_2_CH_2_), 63.0 (C-6), 55.6 (*C*-α), 48.2 (*C*H_2_-CH_Fm_), 40.3 (man-OCH_2_*C*H_2_), 39.3 (C-β), 37.6 (CH_2_-*C*H_Fm_) ppm.

## Supporting Information

File 1 Analytical material: NMR and mass spectra of products **3-dimer**, **6**, **7**, **8**, **9** and **10**.
