# Supplementary material for: S-Fluorenylmethyl protection of the cysteine side chain upon Nα-Fmoc deprotection
Source: Beilstein J Org Chem. 2012 Dec 10;8:2149–55. doi: 10.3762/bjoc.8.242 (PMC3555517; doi:10.3762/bjoc.8.242)
Supplement: File 1 — Analytical material: NMR and mass spectra of products 3-dimer, 6, 7, 8, 9 and 10. [file Beilstein_J_Org_Chem-08-2149-s001.pdf]

Supporting Information

for

S-Fluorenylmethyl protection of the cysteine side chain upon *N*$^{\alpha}$-Fmoc deprotection

Johannes W. Wehner and Thisbe K. Lindhorst*

Address: Otto Diels Institute of Organic Chemistry, Christiana Albertina University of Kiel, Otto-Hahn-Platz 3/4, 24098 Kiel, Germany

Email: Thisbe K. Lindhorst* - tklind@oc.uni-kiel.de

* Corresponding author

Analytical material: NMR and mass spectra of products 3-dimer, 6, 7, 8, 9 and 10

L-Cystine-bis[2-(α-D-mannopyranosyloxy)ethyl]amide (3-dimer)

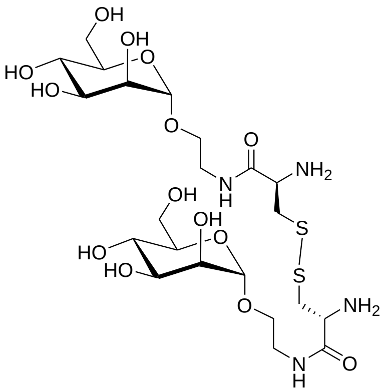

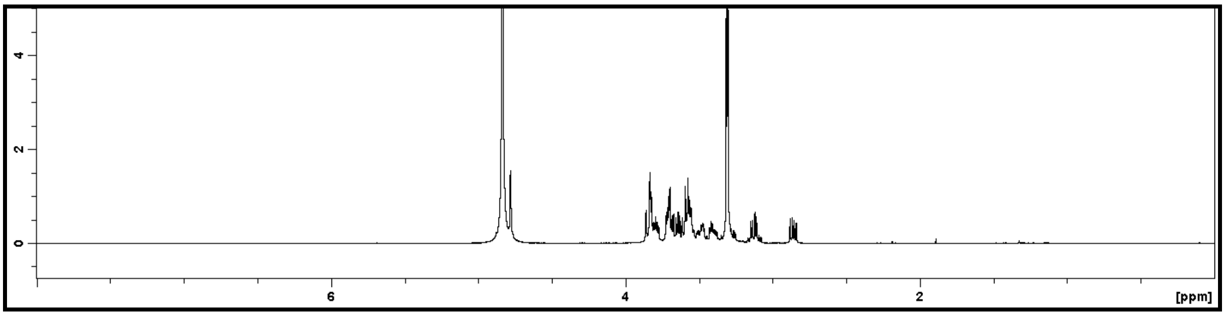

Figure S1: ^{1}H NMR spectrum (500 MHz, CD_3OD) of compound **3-dimer**.

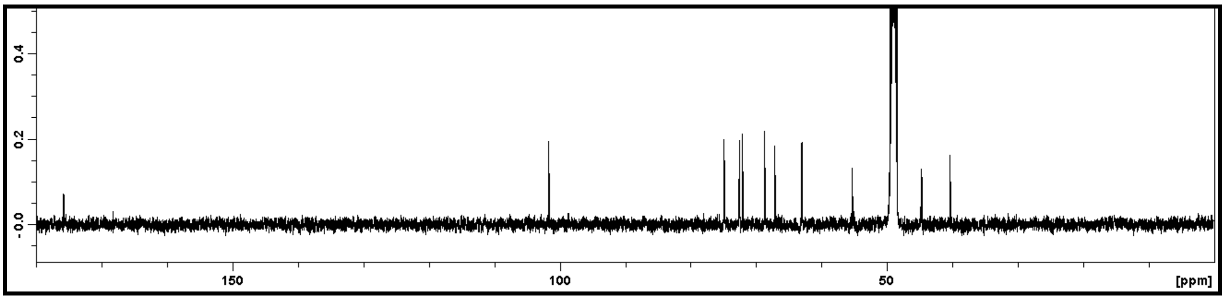

Figure S2: ^{13}C NMR spectrum (125 MHz, CD_3OD) of compound **3-dimer**.

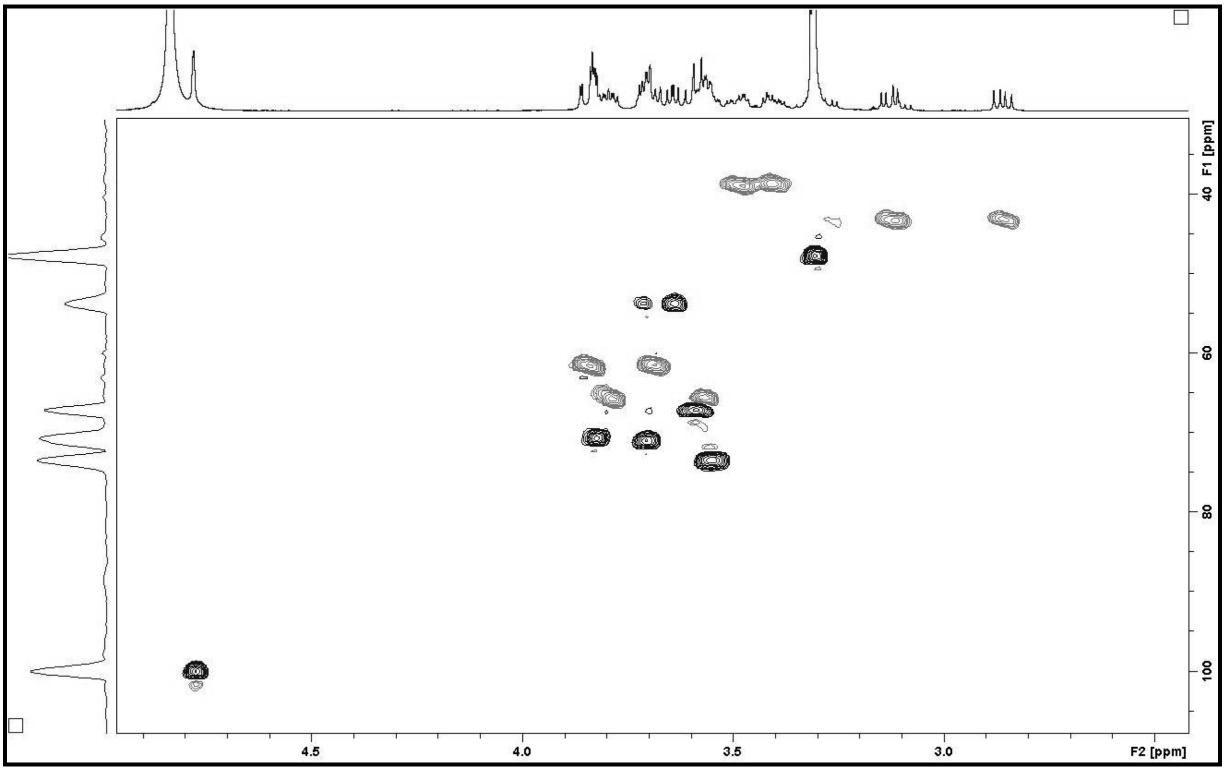

Figure S3: $^{1}H/^{13}C$ HSQC NMR spectrum (125 MHz, CD_3OD) of compound **3-dimer**.

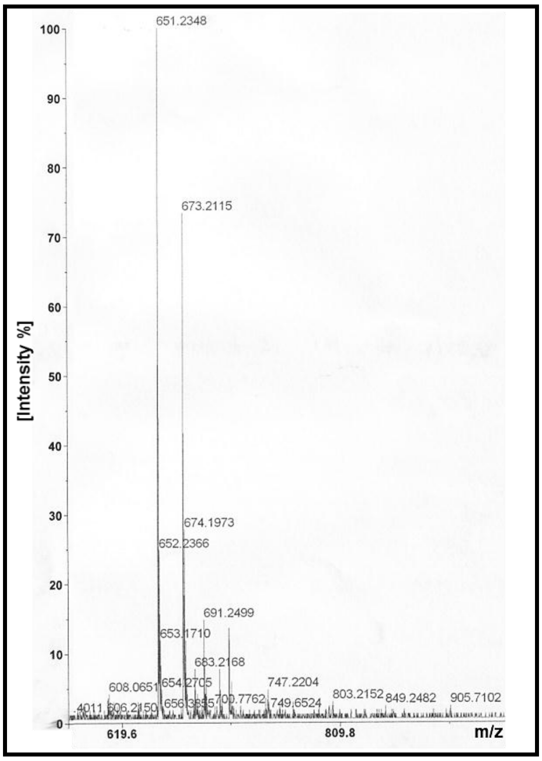

Figure S4: ESI mass spectrum of compound **3-dimer** (*m*/*z* 651.2348 $[M + H]^+$ and *m*/*z* 673.2115 $[M + Na]^+$).

L-Cysteine-[2-(2,3,4,6-tetra-*O*-acetyl-α-D-mannopyranosyloxy)ethyl]amide (6)

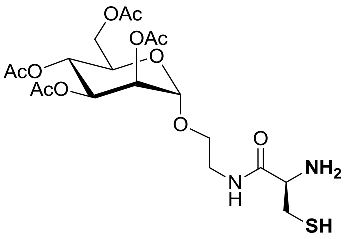

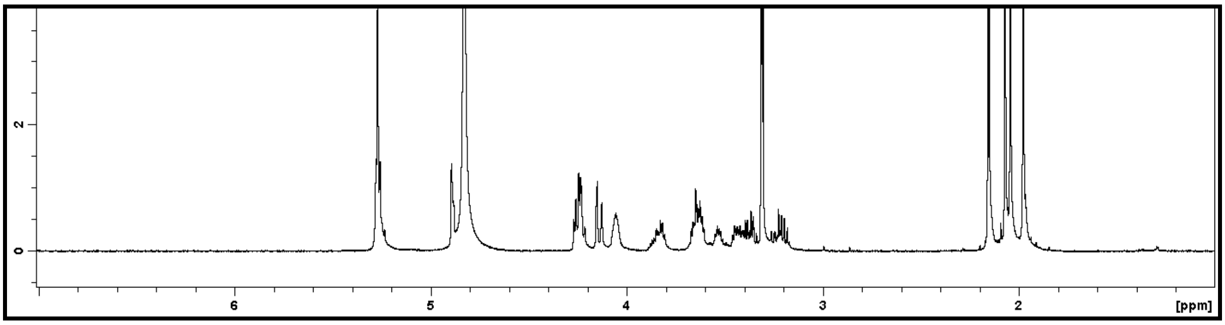

Figure S5: ^{1}H NMR spectrum (500 MHz, CD_3OD) of compound **6**.

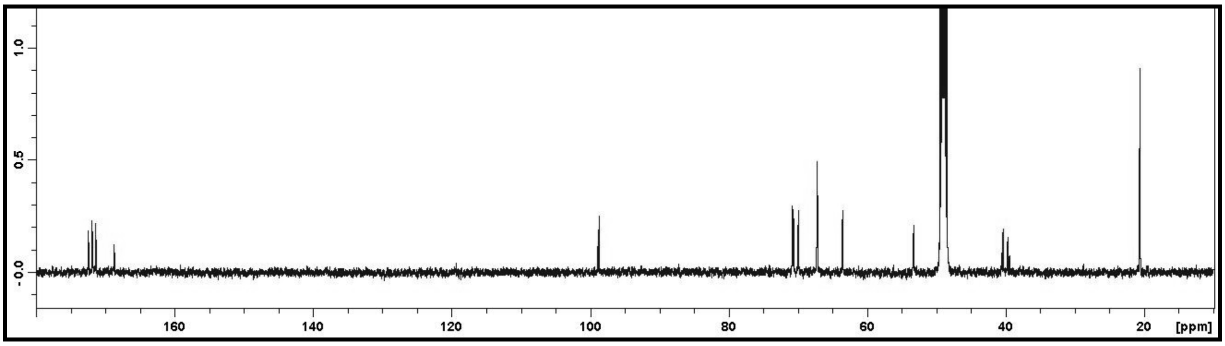

Figure S6: ^{13}C NMR spectrum (125 MHz, CD_3OD) of compound **6**.

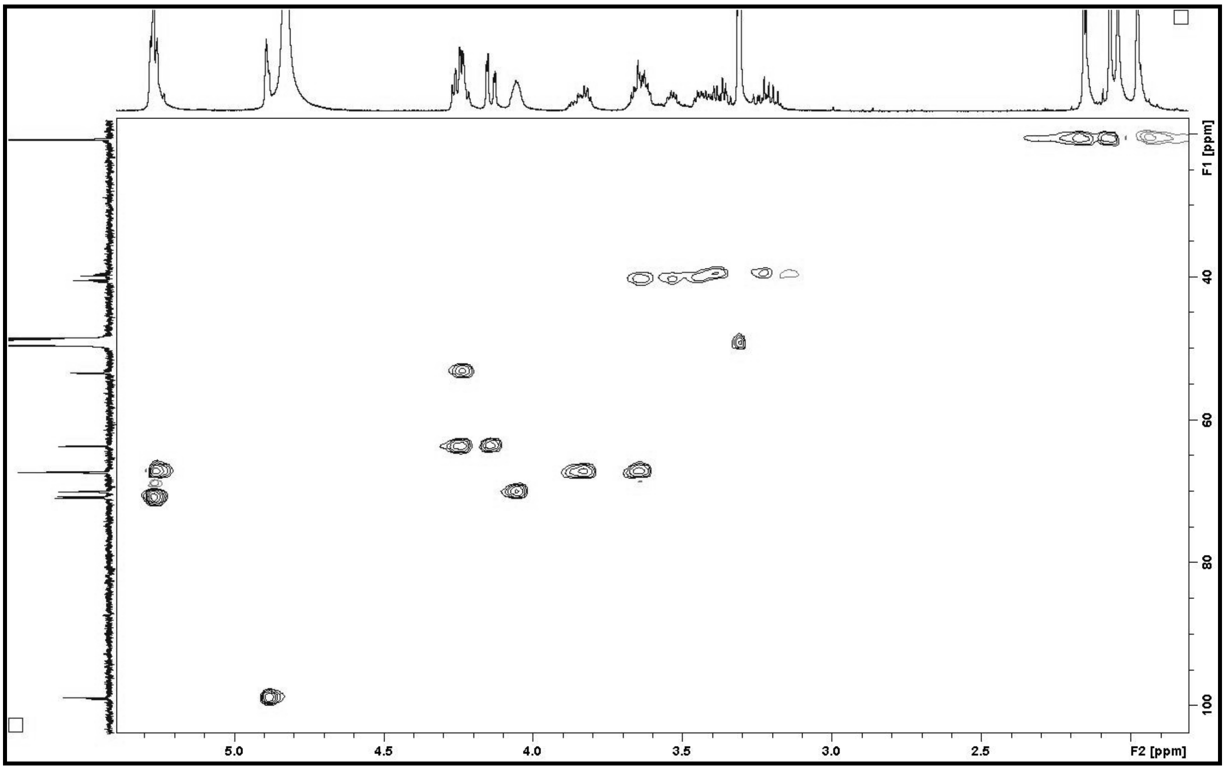

Figure S7: $^{1}H/^{13}C$ HSQC NMR spectrum (125 MHz, CD_3OD) of compound **6**.

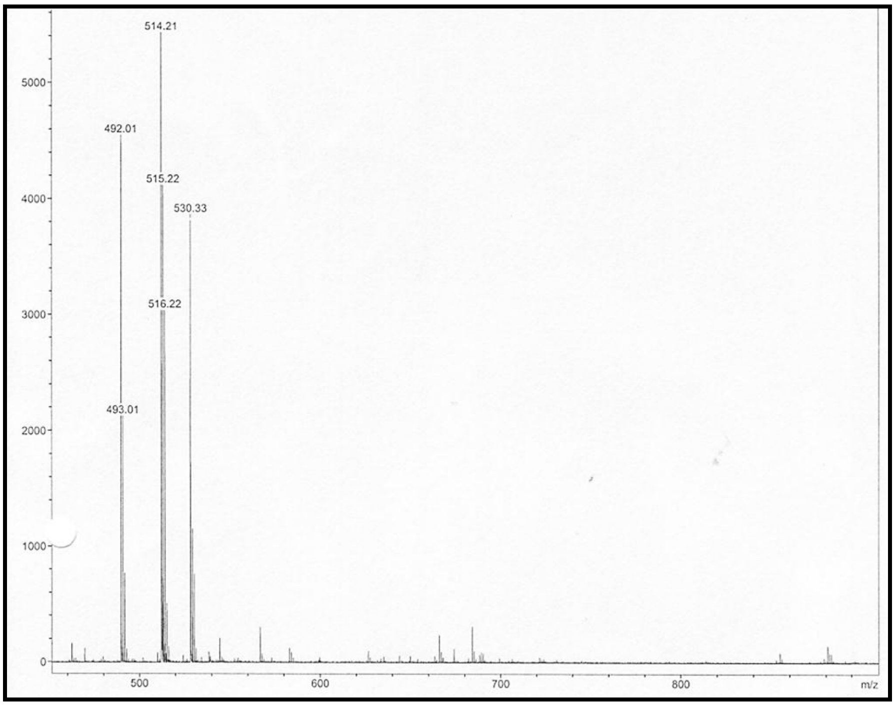

Figure S8: MALDI-TOF mass spectrum of compound **6** (*m*/*z* 492.01 $[M + H]^+$, *m*/*z* 514.21 $[M + Na]^+$ and *m*/*z* 530.33 $[M + K]^+$).

N-(Fluoren-9-ylmethoxycarbonyl)-L-cysteine-[2-(2,3,4,6-tetra-O-acetyl-α-D-mannopyranosyloxy)ethyl]amide (7)

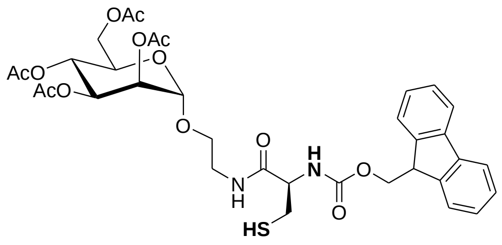

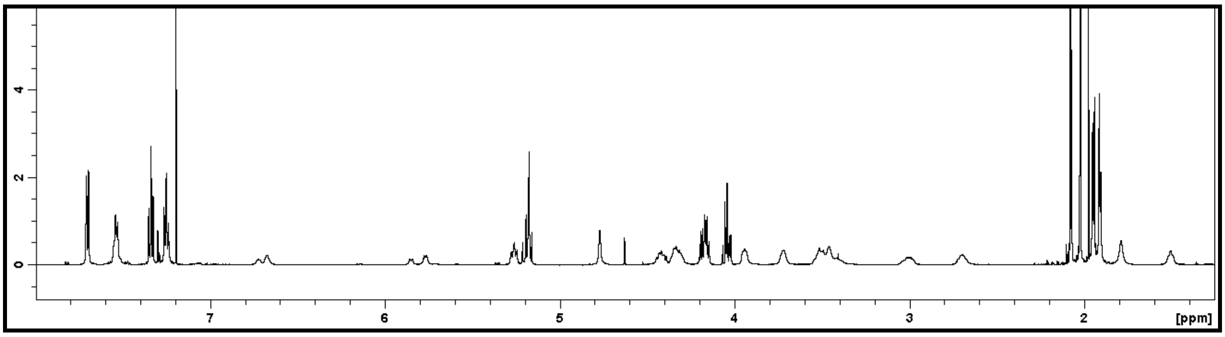

Figure S9: ^{1}H NMR spectrum (600 MHz, $CDCl_3$) of compound **7**.

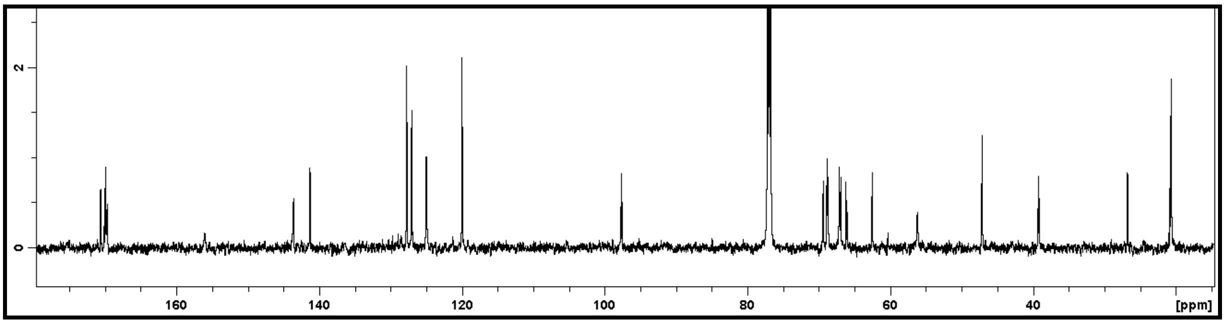

Figure S10: ^{13}C NMR spectrum (150 MHz, $CDCl_3$) of compound **7**.

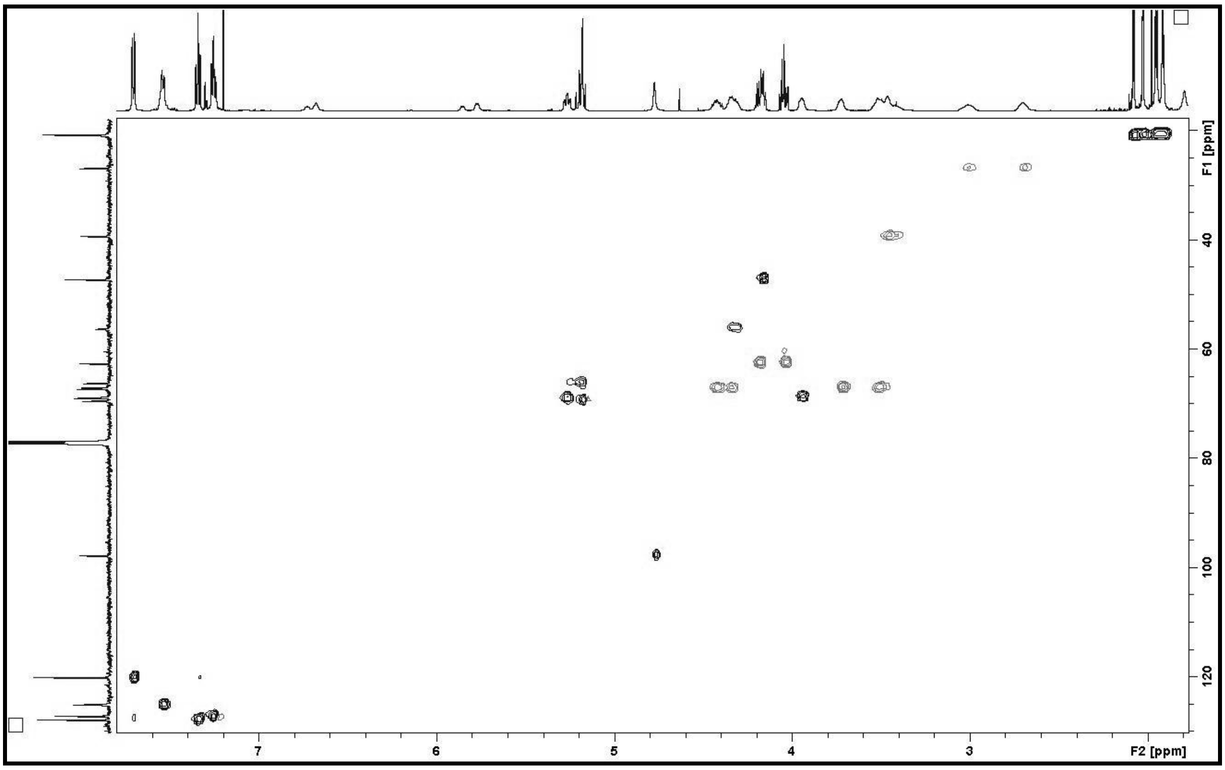

Figure S11: $^{1}H/^{13}C$ HSQC NMR spectrum (150 MHz, $CDCl_3$) of compound **7**.

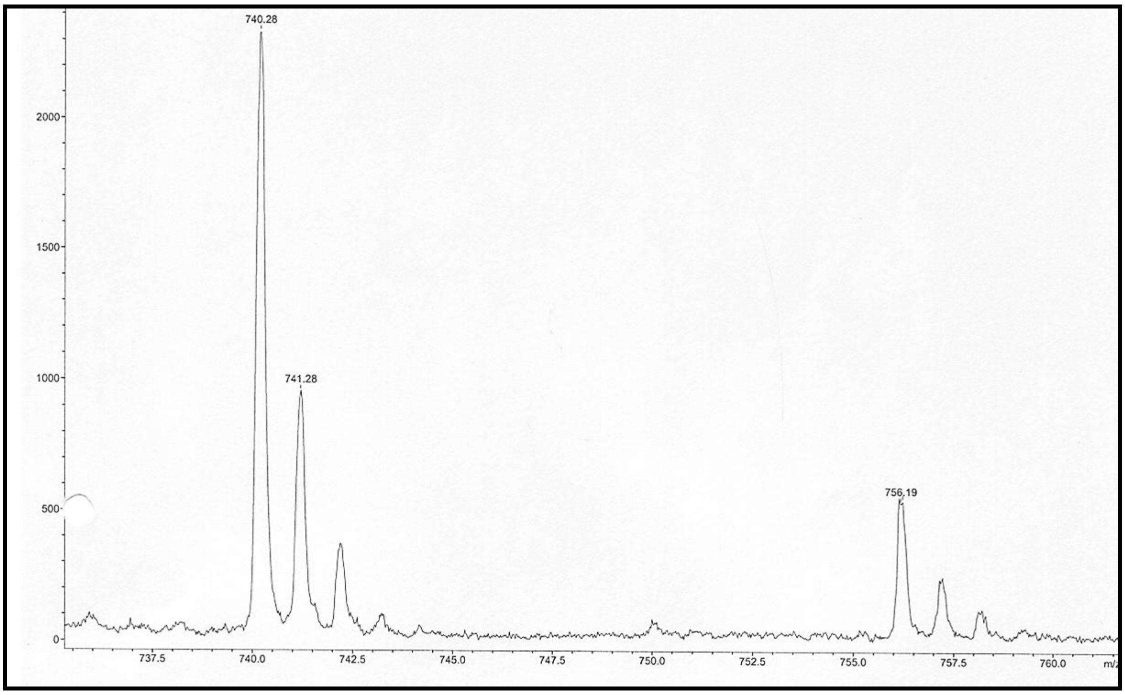

Figure S12: MALDI-TOF mass spectrum of compound **7** (*m*/*z* 740.28 $[M + Na]^+$ and *m*/*z* 756.19 $[M + K]^+$).

***S*-(Fluoren-9-yl)-L-cysteine-[2-(2,3,4,6-tetra-*O*-acetyl-α-D-mannopyranosyloxy)ethyl]amide (8)**

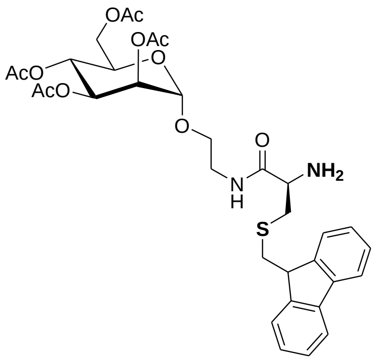

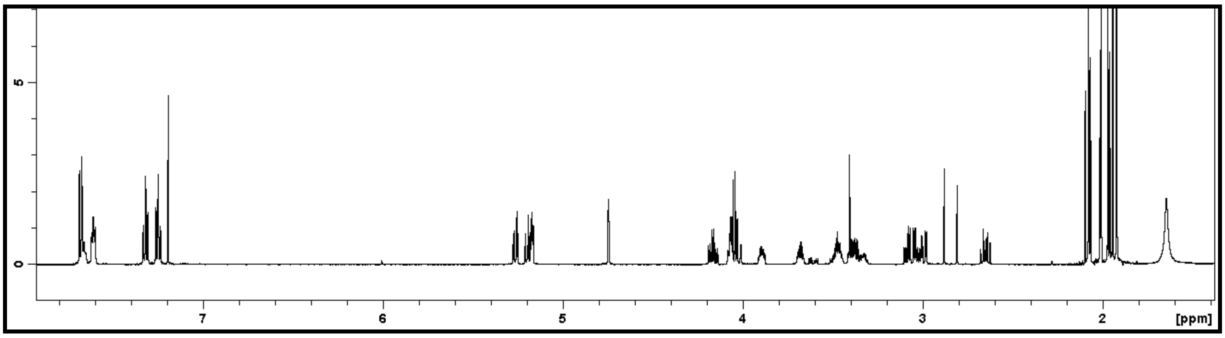

Figure S13: ^{1}H NMR spectrum (600 MHz, $CDCl_3$) of compound **8**.

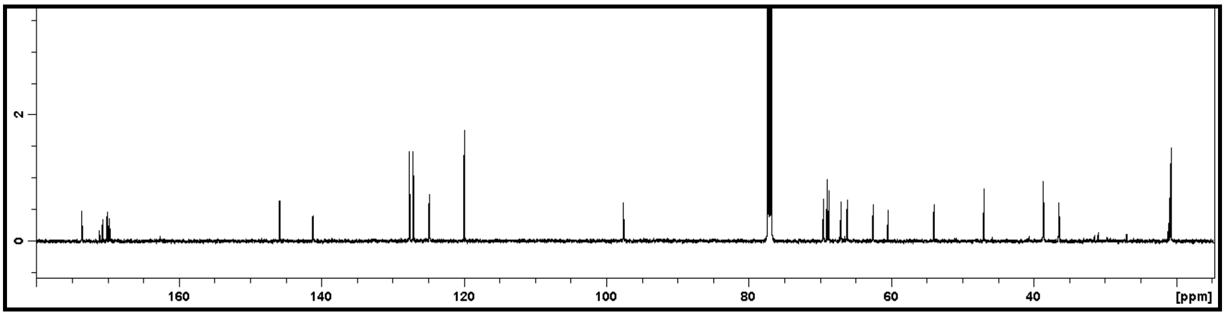

Figure S14: ^{13}C NMR spectrum (150 MHz, $CDCl_3$) of compound **8**.

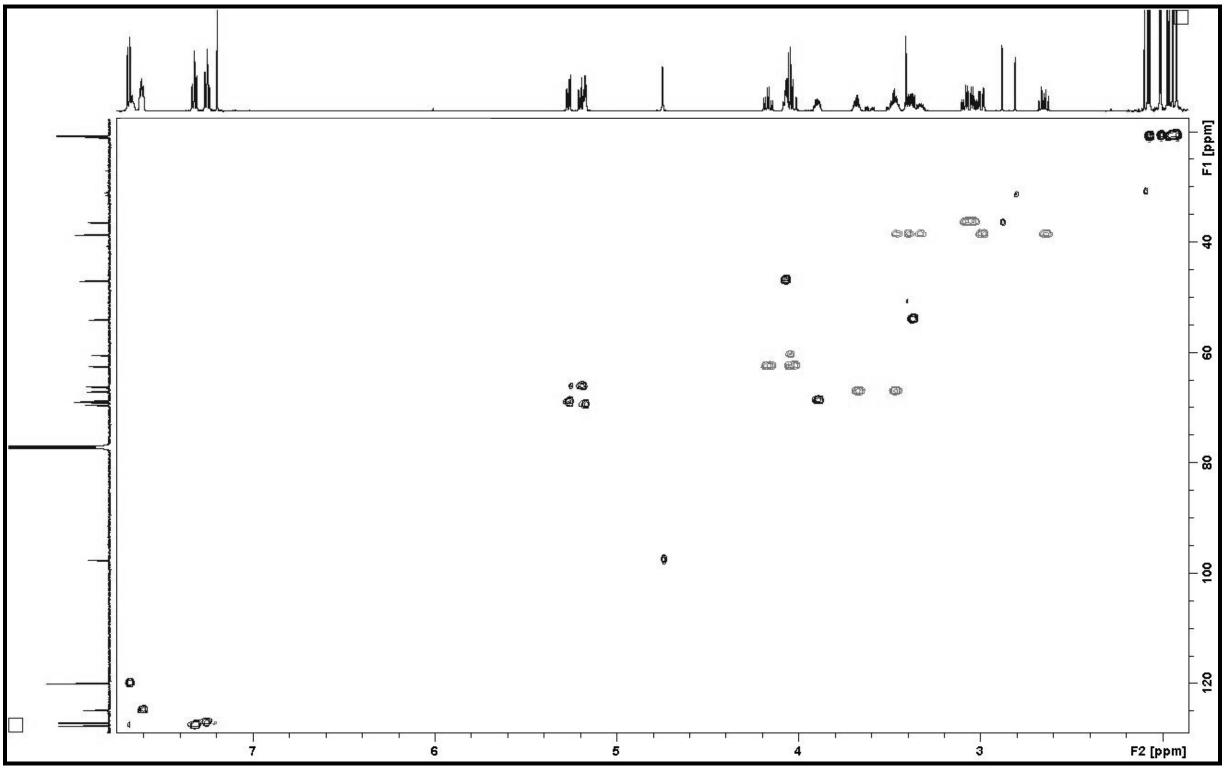

Figure S15: $^{1}H/^{13}C$ HSQC NMR spectrum (150 MHz, $CDCl_3$) of compound **8**.

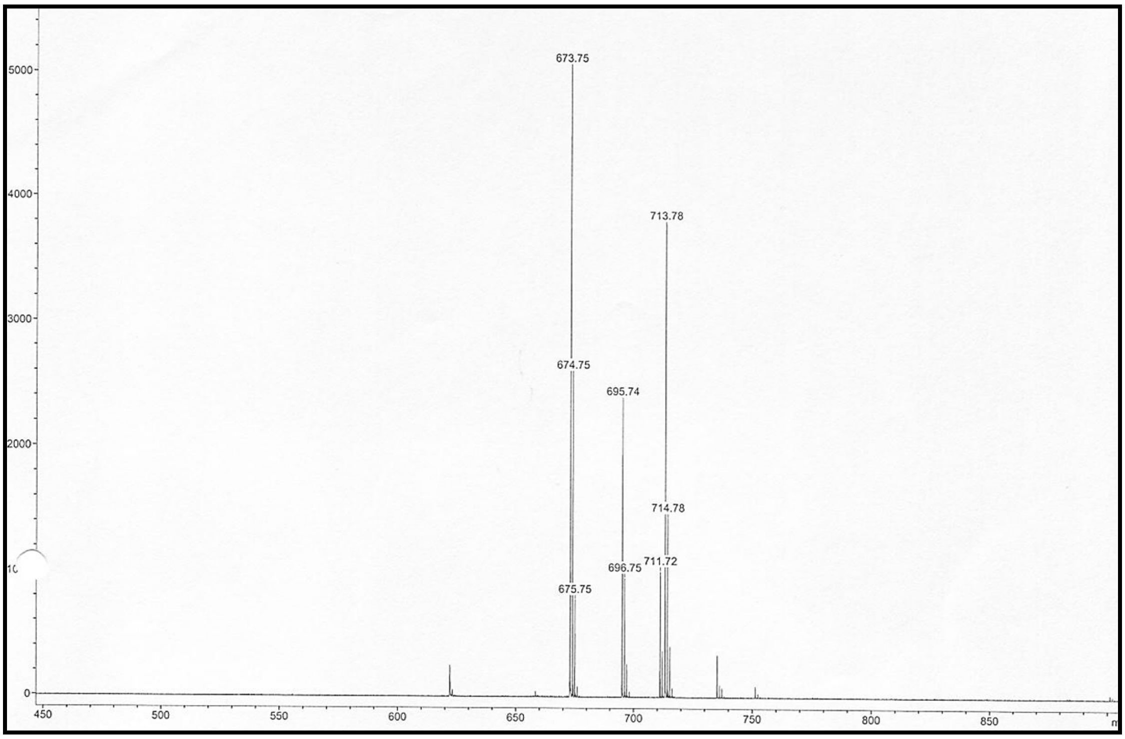

Figure S16: MALDI-TOF mass spectrum of compound **8** (*m*/*z* 673.75 $[M + H]^+$, *m*/*z* 695.74 $[M + Na]^+$ and *m*/*z* 713.78 $[M + K]^+$).

S-(Fluoren-9-yl)-L-cysteine-[2-(α-D-mannopyranosyloxy)ethyl]amide (9)

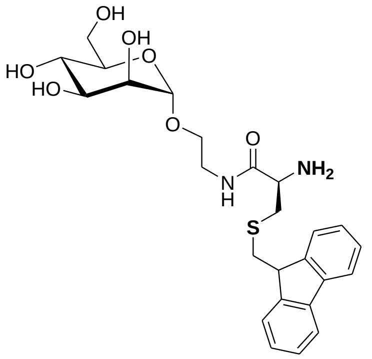

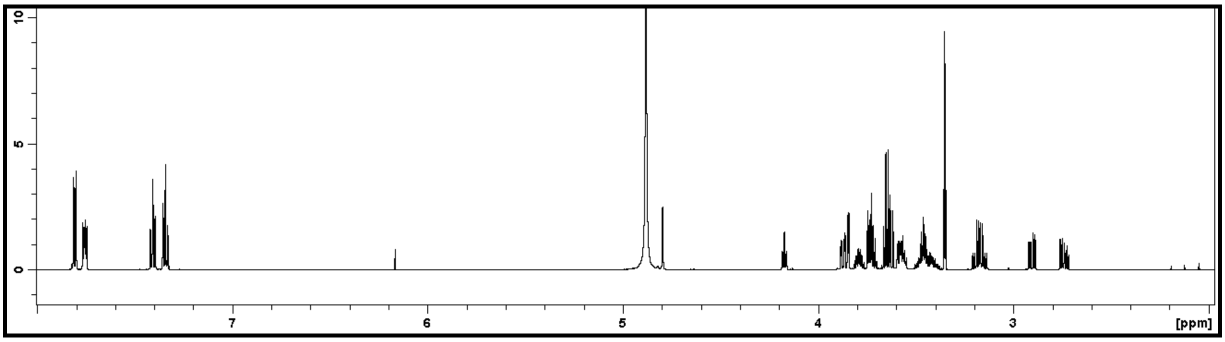

Figure S17: ^{1}H NMR spectrum (600 MHz, CD_3OD) of compound **9**.

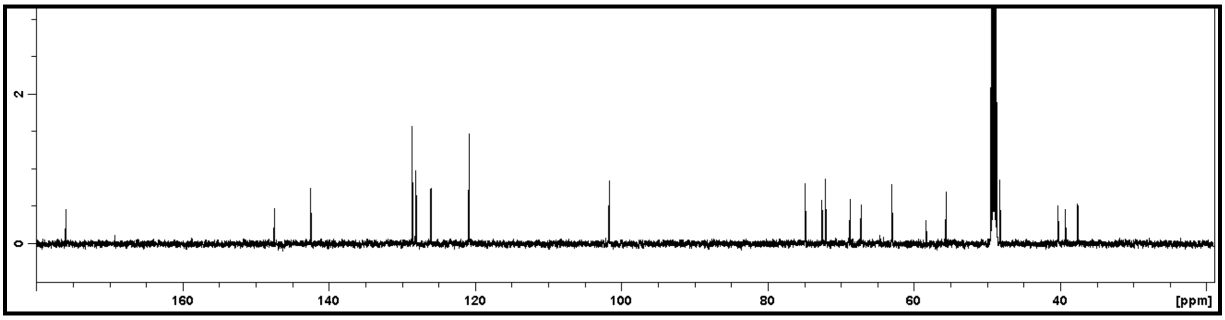

Figure S18: ^{13}C NMR spectrum (150 MHz, CD_3OD) of compound **9**.

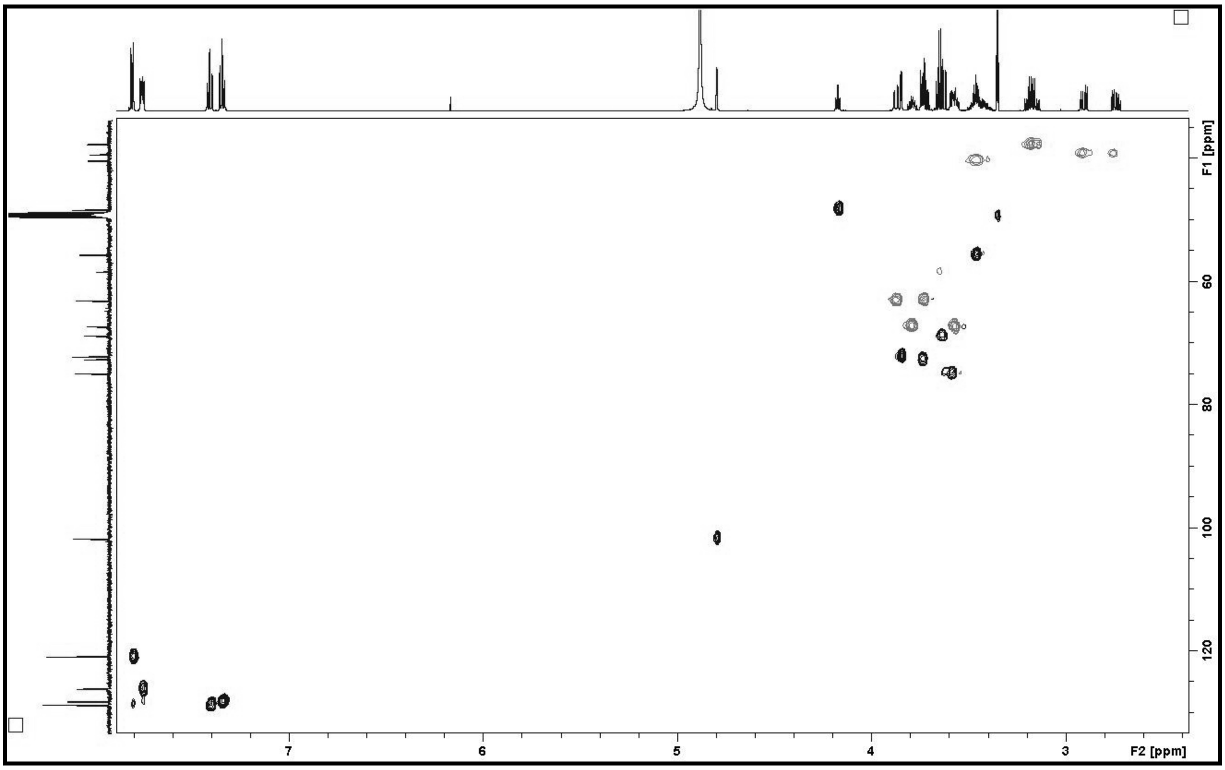

Figure S19: $^{1}H/^{13}C$ HSQC NMR spectrum (150 MHz, CD_3OD) of compound **9**.

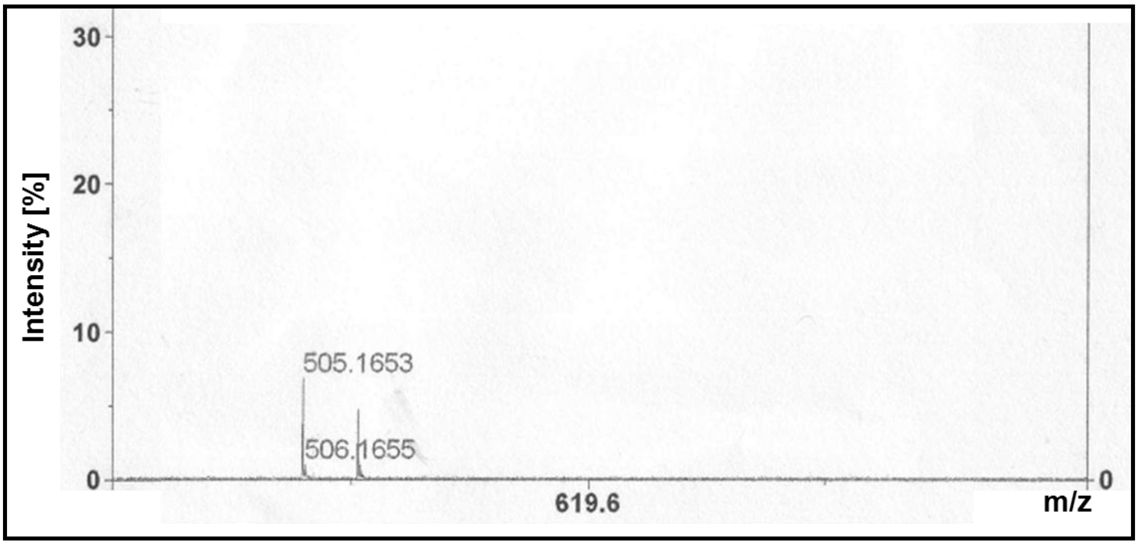

Figure S20: ESI mass spectrum of compound **9** (*m*/*z* 505.1653 $[M + H]^{+}$).

N-(Fluoren-9-ylmethoxycarbonyl)-S-(fluoren-9-yl)-L-cysteine-[2-(2,3,4,6-tetra-O-acetyl-α-D-mannopyranosyloxy)ethyl]amide (10)

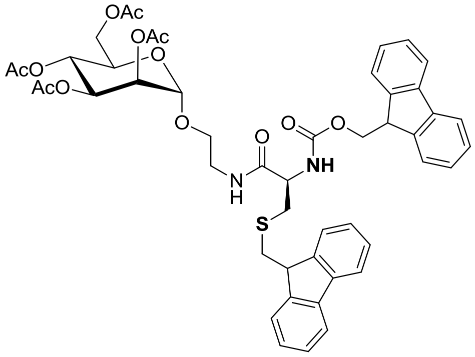

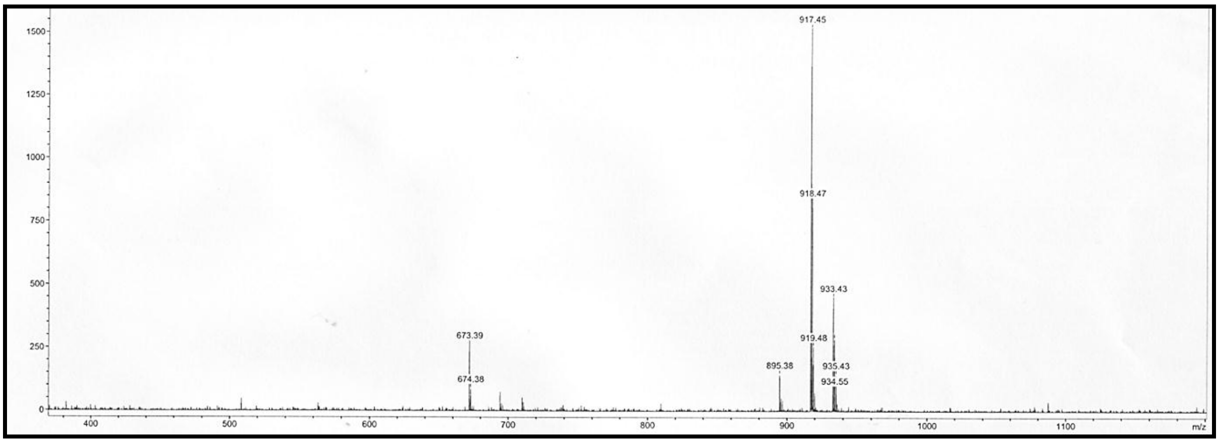

Figure S21: MALDI-TOF mass spectrum of compound **10** (*m*/*z* 895.38 [M + H]$^+$, *m*/*z* 917.45 [M + Na]$^+$ and *m*/*z* 933.43 [M + K]$^+$); *m*/*z* 673.39 is caused by the loss of the *N*-Fmoc-protecting group during ionization.
